# Long-acting CCK analogue NN9056 lowers food intake and body weight in obese Göttingen Minipigs

**DOI:** 10.1038/s41366-019-0386-0

**Published:** 2019-06-07

**Authors:** Berit Ø. Christoffersen, Rikke Bjerring Skyggebjerg, Anne Bugge, Rikke Kaae Kirk, Bill Vestergaard, Henriette Kold Uldam, Johannes Josef Fels, Charles Pyke, Ulrich Sensfuss, Annika Sanfridson, Trine Ryberg Clausen

**Affiliations:** 1 Global Drug Discovery, Novo Nordisk A/S, Novo Nordisk Park, 2760 Måløv, Denmark; 2 Global Research Technologies, Novo Nordisk A/S, Novo Nordisk Park, 2760 Måløv, Denmark

**Keywords:** Drug discovery, Endocrinology, Preclinical research

## Abstract

**Background/Objectives:**

Cholecystokinin (CCK) is a regulator of appetite and energy intake in man. The aim of this study was to determine the effect of NN9056, a long-acting CCK-1 receptor-selective CCK analogue, on food intake and body weight (BW) in obese Göttingen Minipigs.

**Subjects/Methods:**

Tolerability of NN9056 and acute effects on food intake, pancreas histology, amylase and lipase levels were assessed in lean domestic pigs in doses up to 100 nmol/kg (*n* = 3–4). Subsequently, obese Göttingen Minipigs were treated subcutaneously (s.c.) once daily for 13 weeks with vehicle, NN9056 low dose (regulated from 5 to 2 nmol/kg) or NN9056 high dose (10 nmol/kg) (*n* = 7–8). Food intake was measured daily and BW twice weekly. At the end of the treatment period, an intravenous glucose tolerance test (IVGTT) and a 24-h exposure profile was obtained. Data are mean ± SD.

**Results:**

The acute studies in domestic pigs showed significant and dose-dependent effect of NN9056 on food intake, acceptable tolerability and no histopathological signs of pancreatitis. Sub-chronic treatment in obese Göttingen Minipigs was also well tolerated and accumulated food intake was significantly lower in both treated groups compared to vehicle, with no significant difference between the dose levels of NN9056 (41.8 ± 12.6, 51.5 ± 13.8 and 86.5 ± 19.5 kg in high-dose, low-dose and vehicle groups, respectively, *p* = 0.012 and *p* < 0.0001 for low and high dose vs. vehicle, respectively). Accordingly, there was a weight loss in both treated groups vs. a weight gain in the vehicle group (−7.2 ± 4.6%, −2.3 ± 3.2% and 12.3 ± 3.9% in the high-dose, low-dose and vehicle groups, respectively, *p* < 0.0001 for both vs. vehicle). IVGTT data were not significantly different between groups.

**Conclusion:**

NN9056, a long-acting CCK-1 receptor-selective CCK analogue, significantly reduced food intake and BW in obese Göttingen Minipigs after once daily s.c. dosing for 13 weeks.

## Introduction

Obesity is an increasing problem worldwide, with a great unmet medical need [1]. Cholecystokinin (CCK) is a gastrointestinal hormone that could potentially be used as an anti-obesity drug, as evidenced by the lowering of food intake observed after administration to rodents [2, 3], pigs [4], monkeys [5] and both lean and obese humans [6, 7]. CCK exists in a number of biologically active forms [8], is mainly secreted from I cells in the upper gastrointestinal tract in response to a meal [9] and has a circulating half-life of <5 min [10]. There are two CCK receptors, the CCK-1 receptor and the CCK-2 receptor [11]. The CCK-1 receptor is mainly expressed in the periphery in relation to the gastrointestinal tract, and activation of this receptor induces stimulation of gallbladder contraction, pepsinogen, bile and pancreatic enzyme secretion, relaxation of the sphincter of Oddi, inhibition of gastric emptying and intestinal mobility, and suppression of food intake [12–14]. The CCK-2 receptor is expressed in the stomach where activation stimulates gastric acid secretion, in the brain where activation is associated with anxiety and panic attack [11, 12] and on the thyroid c cells mediating calcitonin release upon stimulation [15].

The encouraging weight-modulating effects of sub-chronic CCK-1 receptor agonism observed pre-clinically [3, 16] have to date not been reproduced in the clinic. The small-molecule CCK-1 receptor agonist, GI181771X, was explored as an anti-obesity drug, but failed to induce weight loss in obese humans after 24 weeks of treatment [17]. Sub-optimal exposure levels and lack of full-day coverage might explain the lack of efficacy. Thus, a peptide-based approach, with a half-life-extending acylation or PEGylation, could be more attractive compared to a small-molecule approach, due to better pharmacokinetic (PK) properties with continuous exposure. Further, higher specificity and fewer off-target effects would be expected with a peptide approach.

Potential safety concerns from treatment with CCK or CCK analogues are pancreatitis mediated by pancreatic and/or vagal CCK-1 receptors, and anxiety mediated by central CCK-2 receptors. The risk of anxiety may be mitigated by a compound with high selectivity towards the CCK-1 receptor, whereas CCK-1 receptor-mediated pancreatitis remains a risk. However, the prevalence of CCK-1 receptors on the pancreatic acinar cells is species dependent, potentially leading to species differences in the development of pancreatitis as reviewed by Myer et al. [18]. Rodents and dogs are prone to develop pancreatitis after CCK receptor activation [18, 19], which may be due to a high level of CCK-1 receptor expression in the exocrine pancreas [20]. In contrast, humans and non-human primates have very low expression of CCK-1 receptors [21, 22], indicating a low risk of CCK-induced pancreatitis.

Based on the available data, it was hypothesised that treatment with a long-acting CCK-1 receptor-selective analogue with 24-h plasma exposure coverage would result in a significant and clinically relevant reduction in food intake and body weight (BW) without causing pancreatitis in humans. The acylated CCK analogue NN9056 is a highly CCK-1 receptor-selective compound with a terminal plasma half-life of approximately 110 h in young lean minipigs [23]. Due to the high expression of CCK-1 receptors in the pancreas, rodents and dogs were deselected as preclinical efficacy models, leaving pigs and non-human primates as relevant and well-characterised obesity models. For ethical reasons, the obese Göttingen Minipig model was chosen. Female Göttingen Minipigs readily develops obesity when fed *ad libitum* on chow, and translational value with respect to weight loss has previously been shown with liraglutide [24].

The aim of the present study was to evaluate the effect of NN9056, a novel long-acting and selective CCK-1 receptor agonist, on food intake and BW in obese Göttingen Minipigs. Prior to the sub-chronic study, CCK-1 receptor selectivity, binding and activation by NN9056 were demonstrated in vitro to qualify the pig as a pharmacological model, and acute tolerability and PK were assessed in vivo to enable dose setting in the sub-chronic study.

## Methods

### In vitro assays

#### CCK receptor binding and activation studies

CCK analogue NN9056 and sulfated native human CCK-8 was synthesised at Novo Nordisk A/S [23]. All experiments were carried out essentially as previously described [25]. In brief, pig or human CCK receptors were over-expressed in COS-7 cells that were subsequently used for receptor-binding studies or receptor activation studies. Receptor binding was determined using a scintillation proximity assay (SPA) where displacement of radiolabelled CCK-8 tracer by ligands was determined as a reduction in light emission from the scintillant inside the SPA beads. Receptor activation was assessed using the IP-One homogeneous time-resolved fluorescence assay (Cisbio), where increasing levels of the endogenous second messenger inositol 1 monophosphate (IP1) is measured as a reduction in fluorescence resonance energy transfer between Tb-cryptate-conjugated anti-IP1 antibody and d2-conjugated IP1. Relative receptor affinity (IC_50_ (half-maximal inhibitory concentration)) and potency (EC_50_ (half-maximal effective concentration)) values were calculated in GraphPad Prism v 6.0 (GraphPad software, La Jolla, CA, USA) by nonlinear regression analysis of sigmoidal dose–response curves with constraint on the bottom value to equal that of the reference compound; CCK-8. For the IP-One data, the Hill slope was constrained to be <0. All studies were performed in technical duplicate and repeated independently three times (*n* = 3).

#### CCK-1 receptor in situ hybridisation

Tissue was fixed in buffered formalin (10% NBF) and paraffin-embedded (FFPE) according to standard protocols. Tissue blocks were cut at 4.5 µm and in situ hybridisation (ISH) was applied using a manual RNAscope^®^ single-plex protocol according to the manufacturer’s instructions (322310-QKG, Advanced Cell Diagnostics, Hayward, CA, USA). Sections were hybridised with positive control probes PolR2A (polymerase II subunit A, RNAscope^®^ probe cat. nos. 3110451, 3124711 and 312481, Advanced Cell Diagnostics, Hayward, CA, USA) and negative control probe DapB (dihydrodipicolinate reductase, RNAscope^®^ probe cat. no. 310043, Advanced Cell Diagnostics, Hayward, CA, USA). Two consensus probes for the CCK1R were used, one covering mouse and rat (RNAscope^®^ probe cat. no. 411521, Advanced Cell Diagnostics, Hayward, CA, USA) and the other covering human, dog and pig (RNAscope^®^ probe cat. no. 411511, Advanced Cell Diagnostics, Hayward, CA, USA).

### In vivo studies

All in vivo studies were conducted in accordance with national regulations in Denmark, which are fully compliant with internationally accepted principles for the care and use of laboratory animals, and with animal experimental licenses granted by the Danish Ministry of Justice. Power calculations for each study type are described in Supplementary Table S1.

#### Statistical analysis

All statistical analyses were performed in GraphPad Prism (GraphPad Prism version 6.07 for Windows, GraphPad Software, San Diego, CA, USA) as described below under the individual studies. Normal distribution was assumed for all parameters. When using one-way analysis of variance (ANOVA), a Brown–Forsythe test was used to compare variances in the three groups. In case of unequal variance, a Kruskal–Wallis test followed by Dunn’s multiple comparison test was performed. *P* values below 0.05 were considered statistically significant. Significance levels *p* < 0.001 (***), 0.01 (**) and 0.05 (*) are indicated in relevant figures.

#### Dosing solutions

In the tolerability study in lean domestic pigs (Landrace, Yorkshire Duroc (LYD)), the dosing solutions consisted of 50 mM phosphate, 70 mM sodium chloride and 0.05% polysorbate 80 with pH adjusted to 7.4 and NN9056 in concentrations of 0, 540 and 5458 nmol/ml. In the acute effect study in lean LYD pigs and in the two studies in obese Göttingen Minipigs, dosing solutions consisted of 8 mM phosphate, 184 mM propylene glycol and 58 mM phenol with pH adjusted to 7.4 and NN9056 in concentrations of 0, 424, 751, 1465, 1860 and 2994 nmol/ml in the LYD study, 1860 nmol/ml in the Göttingen Minipig PK study and 0, 1902/2198 and 9727 nmol/ml in the Göttingen Minipig effect study.

#### Measurement of food intake in lean LYD pigs

Lean, female LYD pigs (Gundsoegaard, Roskilde, Denmark), approximately 3 months of age weighing approximately 30–35 kg, were used. The animals were fed *ad libitum* with Danish Top S1 611 (Danish Agro A.m.b.a., Karise, Denmark) and had free access to water. Daily food intake was monitored online by logging the weight of fodder every 15 min (Mpigwin, Ellegaard Systems, Faaborg, Denmark). Food intake was monitored for up to 3 days prior to compound administration and up to 4 days after administration.

#### Acute tolerability and adverse pancreas effects in lean LYD pigs

Animals were allocated into three groups of four animals based on baseline food intake and BW, and dosed subcutaneously (s.c.) with 0.02 ml/kg to achieve a nominal dose of either 0, 10 or 100 nmol/kg. At pre-dose and at 48 h post-dose, a ethylenediaminetetraacetic acid (EDTA)-stabilised blood sample was taken and plasma concentrations of NN9056, pancreatic lipase, pancreatic α-amylase and total bile acids (TBAs) were measured as described in Supplementary Table S2. Pigs were then euthanised by intramuscular (i.m.) injection of 1 ml/kg Zoletil mixture (Supplementary Table S3), followed by exsanguination by cutting the large arteries to the front legs. Pancreatic tissue was sampled 48 h after dosing from one pig dosed with 10 nmol/kg and four pigs dosed with 100 nmol/kg NN9056. Tissue samples were fixed in 10% buffered formalin for approximately 24 h, processed in a tissue processor (Leica ASP300S, Triolab, Brøndby, Denmark), paraffin embedded, cut at 3 µm thickness and stained with haematoxylin and eosin (Merck KGaA, Darmstadt, Germany).

Delta values of lipase, α-amylase and TBA were compared between the groups using one-way ANOVA with Dunnett’s multiple comparison post-test. Daily food intake was compared to vehicle using repeated-measures two-way ANOVA, with “day” and “treatment” as explanatory variables followed by Bonferroni’s post-test. Pancreas sections were qualitatively evaluated for signs of pancreatitis.

#### Acute effect on food intake in lean LYD pigs

Animals were allocated into one group of three animals (vehicle) and four groups of four animals based on baseline food intake and BW. In the morning on day 0, the animals were given a s.c. dose of NN9056 or vehicle, and food intake was followed for 4 days. The dosing volume used was 0.025 ml/kg to achieve nominal doses of 0, 10, 20, 40 and 80 nmol/kg. In the end of each study, the animals were anaesthetised with Zoletil mixture given i.m. (Supplementary Table S3), and an EDTA-stabilised blood sample was taken from the saphenous vein, before euthanasia. Resulting plasma was stored at −20 °C until analysis of plasma NN9056 content as described in Supplementary Table S2.

Statistical comparison of daily food intake between the treated groups and vehicle was done using repeated-measures two-way ANOVA, with “day” and “treatment” as explanatory variables, followed by Bonferroni’s post-test comparing each dosing group to the vehicle group.

#### Studies in obese Göttingen Minipigs

These studies were performed in obese, female, ovariectomised (OVX) Göttingen Minipigs (Ellegaard Göttingen Minipigs A/S, Dalmose, Denmark). Obesity had been induced by feeding increasing amounts of SDS minipig diet (SDS, Essex, UK) or Altromin 9023 (Brogaarden, Lynge, Denmark) for at least 10 months before study start. During the studies, the pigs were fed with Altromin 9023 (Brogaarden, Lynge, Denmark) (restricted in the PK study and *ad libitum* in the effect study) and had free access to water.

#### PK evaluation in obese Göttingen Minipigs

The study was performed in three obese Göttingen Minipigs, approximately 2 years of age at study start and with a mean BW (±SD) of 74.5 ± 8.3 kg (range: 66.7–83.3 kg). The pigs were instrumented with permanent central venous catheters in the cranial caval vein (Cook, C-TPNS-6.5-90-REDO, 6.5 French, Cook Medical, Bjæverskov, Denmark) 3 weeks before study start as previously described [26].

The test compound was given as a s.c. injection (2.5 µL/kg to achieve a nominal dose of 5 nmol/kg) using a NovoPen Echo^®^4. Blood samples were collected in EDTA-coated tubes at the following time points relative to dosing: predose, 0.083, 0.25, 0.5, 0.75, 1, 1.5, 2, 3, 4, 6, 8, 10, 24, 30, 48, 72, 96, 120, 168, 192, 216, 240, 264, 288, 336, 360, 384, 408, 432 and 456 h. The resulting plasma was analysed for NN9056 plasma concentration as described in Supplementary Table S2.

One-compartmental PK modelling was performed in Phoenix WinNonlin Professional 6.4 (Pharsight, Mountain View, CA, USA). Data analysis was performed using individual plasma concentration–time values. The given mean values are all geometric means.

#### Sub-chronic effects on food intake and BW in obese Göttingen Minipigs

The study was performed in 30 obese Göttingen Minipigs, approximately 18 months of age at study start. After a baseline period of 4 weeks with *ad libitum* feeding and no treatment, the animals were randomised into three groups. Group 1 was treated s.c. with vehicle (*n* = 8) once daily (QD), Group 2 (*n* = 7) was treated s.c. with NN9056 QD at a low dose level (regulated from 5 to 2 nmol/kg), Group 3 (*n* = 7) was treated s.c. with NN9056 QD at a high dose level (10 nmol/kg). All groups were treated for 13 weeks, whereafter they were subjected to a 3-week wash-out period.

On study days −8 to −5, 50–51 and 78–80, the animals were fasted and anaesthetised for body composition evaluation by dual-energy x-ray absorptiometry scanning. Unfortunately, the measurements were invalid (since a decrease in body fat percentage in the vehicle group was observed, instead of the expected increase typical for this model) and are therefore not included in the Results section, but mentioned here since the food intake data are influenced by the procedure. During the procedure, the animals were anaesthetised with Zoletil mixture i.m. and in addition given an i.m. injection of 0.006–0.01 mL/kg Atropine (1 mg/ml) (Supplementary Table S3).

In the same anaesthesia, on days 78–80, a central venous catheter (BD Careflow 3Fr 200 mm, Argon Medical, TX, USA) was implanted in the jugular vein via an ear vein using a minimally invasive technique. This catheter was used for infusion and for blood sampling.

Measurement of the daily food intake was performed during the entire study period, by logging the weight of fodder online using the MP-2 system (Mbrose Aps, Faaborg, Denmark). BW was obtained twice weekly on a large animal scale during the entire study. Accumulated food intake and BW change from days 1 to 77 were calculated as primary parameters of interest, and statistical comparison between the three groups was done using one-way ANOVA followed by Tukey’s multiple comparisons test. In addition, BW loss from baseline was evaluated by comparing BW on days 1 and 77 within each group using two-way ANOVA followed by Sidak’s multiple comparisons test.

#### Glucose tolerance and plasma parameters in obese Göttingen Minipigs

An intravenous glucose tolerance test (IVGTT) was performed in approx. 18 h fasted animals after 12 weeks of treatment on study days 84–86. An intravenous glucose bolus of 0.3 g/kg (0.6 mL/kg of a sterile 50% glucose solution, glucose 500 g/L; SAD) was given through the central venous catheter. Blood samples for glucose and insulin analysis were collected in EDTA tubes (8 mM) with 30 μl Aprotinin (10,000 KIU/ml, Nordic Pharma, Denmark) added to the tubes, at the following time points in relation to the glucose load: predose 1, pre-dose 2 (just before the glucose bolus), 1, 3, 5, 7, 10, 15, 20, 25, 30, 35, 40, 50 and 60 min. In addition, a fasting plasma sample of 2 ml was obtained on days 87–88 for measurement of fasting plasma glucose, insulin, C-peptide, glucagon, leptin, TBA, triglycerides (TG), total cholesterol (TC), amylase and lipase. These blood samples were collected in tubes containing 50 µl special stabilisation buffer (Supplementary Table S3). The resulting plasma was pipetted on dry ice and stored at −20 °C until analysis. From the IVGTT, AUC_insulin_ from 0 to 60 min and *K*_G_ (= negative slope of the linear regression of the natural logarithm to glucose versus time in the time interval from 5 to 30 min) were calculated. Fasting plasma parameters, *K*_G_ and AUC_insulin_ were compared between the three groups using one-way ANOVA followed by Tukey’s multiple comparisons test.

On the last dosing day (day 91), a 24-h exposure profile was obtained followed by regular blood samples during the 3 weeks wash-out period, and average 24-h steady-state NN9056 plasma concentrations were calculated.

### Analytical procedures

Plasma concentrations of NN9056 were measured using liquid chromatography mass spectrometry. Further details and description of the bioanalysis for the remaining plasma parameters can be found in Supplementary Table S2.

### Data exclusions

Data exclusions are described in Supplementary Table S1.

## Results

### In vitro studies

#### In vitro binding and activity of NN9056 on porcine and human CCK-1 and CCK-2 receptors

Whereas the CCK receptor subtypes share only ~50% homology, they are highly conserved across species. The human and pig orthologues of the CCK-1 and CCK-2 receptors share 83% and 91% amino acid identity, respectively (UniProt).

As summarised in Table 1, the relative affinity of NN9056 towards the porcine and human CCK-1 receptors was highly similar, also in comparison with the reference compound CCK-8. Furthermore, as opposed to the strong binding observed with CCK-8 on porcine and human CCK-2 receptors, no IC_50_ values for NN9056 could be determined at the concentrations tested, indicating high CCK-1 receptor selectivity of NN9056 in both species.

**Table 1:** NN9056 binding and activation of porcine and human CCK-1 and CCK-2 receptors

	CCK-1 receptor				CCK-2 receptor			
	NN9056		CCK-8		NN9056		CCK-8	
Binding	Mean IC_50_ (nM)	95% CI (nM)	Mean IC_50_ (nM)	95% CI (nM)	Mean IC_50_ (nM)	95% CI (nM)	Mean IC_50_ (nM)	95% CI (nM)
Pig	0.123	0.037–0.406	0.199	0.173–0.211	>125^a^	N/A	0.102	0.039–0.262
Human	0.105	0.099–0.111	0.203	0.137–0.302	>125^a^	N/A	1.876	0.823–4.277

Activation	Mean EC_50_ (nM)	95% CI (nM)	Mean EC_50_ (nM)	95% CI (nM)	Mean EC_50_ (nM)	95% CI (nM)	Mean EC_50_ (nM)	95% CI (nM)
Pig	0.205	0.073–0.573	0.221	0.146–0.335	>100^b^	N/A	0.325	0.241–0.438
Human	0.093	0.059–0.147	0.059	0.041–0.085	>100^b^	N/A	0.367	0.300–0.448

Data are presented as mean with 95% CIs, *n* = 3

*CI* confidence interval, *IC*_*50*_ half-maximal inhibitory concentration, *EC*_*50*_ half-maximal effective concentration

^a^Mean IC_50_ is designated as >125 nM because the IC_50_ values from the individual experiments were larger than the highest compound dose tested (125 nM) or could not be determined by GraphPad Prism

^b^Mean EC_50_ is designated as >100 nM because the EC_50_ values from the individual experiments were larger than the highest compound dose tested (100 nM) or could not be determined by GraphPad Prism

Functionally, NN9056 displayed relative potency at picomolar concentrations on both porcine and human CCK-1 receptors, comparable to the activity seen with CCK-8 (Table 1). As expected, CCK-8 also potently activated the CCK-2 receptor, whereas NN9056 activation occurred only weakly at the highest concentration tested (100 nM), again indicating that the CCK-1 receptor selectivity of NN9056 is preserved in both species.

Taken together, these in vitro data indicate that the pig qualifies as a relevant animal model for pharmacological studies of NN9056.

#### ISH for the CCK-1 receptor in mouse, rat, dog, pig and human

The ISH analyses showed that the CCK-1 receptor messenger RNA (mRNA) expression level in the pancreatic acinar cells was species specific, with high expression observed in the mouse and rat, medium expression in the dog and low expression in the pig and human (see Supplementary Fig. S1). The low CCK-1 receptor mRNA expression in the pig is comparable to the level seen in humans, and predicts a low risk of CCK-1 mediated pancreatitis.

### In vivo studies

#### Acute tolerability and adverse pancreas effects in lean LYD pigs

All animals dosed with 100 nmol/kg NN9056 had subdued behaviour 2 h post dosing. Three animals had increased respiration (from 50–60 respirations/min to 80–96 respirations/min), warm skin (no fever) and no appetite for snacks. CCK molecules are known for the ability to cause nausea and vomiting, and the subdued behaviour observed could potentially be a sign of nausea, but since no vomiting occurred no firm conclusion could be drawn regarding this. From 11 h post dosing, none of the animals had any clinical signs. Food intake was significantly reduced (86 ± 4% (corresponding to −1.1 ± 0.1 kg) from 0 to 24 h and 75 ± 3% (corresponding to −0.8 ± 0.03 kg) from 25 to 48 h, compared to vehicle (*p* < 0.001 for both). Plasma concentration of NN9056 was 555 ± 36.4 nM after 48 h in the treated animals.

No clinical signs were observed in animals dosed with 10 nmol/kg NN9056 and food intake was not significantly reduced (22 ± 5% (corresponding to −0.3 ± −0.1 kg) from 0 to 24 h and 33 ± 7% (corresponding to −0.4 ± 0.1 kg) from 25 to 48 h) compared to vehicle. Plasma concentration of NN9056 was 43.0 ± 1.6 nM after 48 h in the treated animals.

Pancreatic tissue from one pig dosed with 10 nmol/kg and four pigs dosed with 100 nmol/kg NN9056 were microscopically evaluated and no histopathological changes suggestive of pancreatitis were observed in any of the samples (data not shown). In all animals exposed to NN9056, pancreatic lipase, pancreatic α-amylase and TBA were measured in plasma pre-dose (basal) and post-dose. No significant differences were seen pre- or post-dose between NN9056-treated animals and vehicle-treated animals (Supplementary Table S4).

#### Acute effect on food intake in lean LYD pigs

**Fig. 1:**
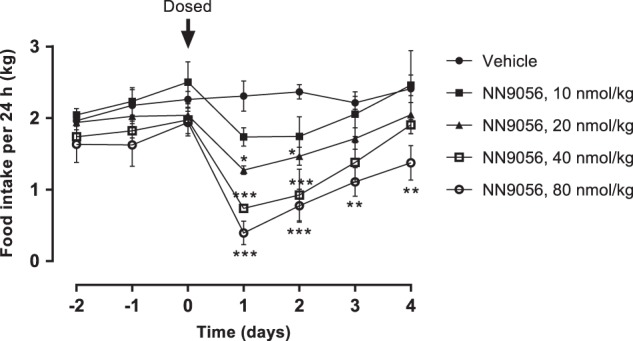
Mean daily food intake (kg) after a single subcutaneous (s.c.) dose of vehicle, 10, 20, 40 and 80 nmol/kg NN9056 in lean LYD pigs. *n* = 3 for vehicle and 40 nmol/kg, *n* = 4 for the others, mean ± SEM. Food intake compared using repeated-measures two-way analysis of variance (ANOVA), with “day” and “treatment” as explanatory variables, followed by Bonferroni’s post-test comparing each dosing group to the vehicle group. *p < 0.05, **p < 0.01, ***p < 0.001

Food intake was dose dependently reduced after a single s.c. dose of 10, 20, 40 or 80 nmol/kg NN9056 (Fig. 1). NN9056 of 20 and 40 nmol/kg significantly reduced food intake for 2 days and of 80 nmol/kg significantly reduced food intake for at least 4 days (Fig. 1). The plasma concentration of NN9056, measured 4 days after dosing, were 22.6 ± 4.5, 39.7 ± 17.1, 86.2 ± 6.9 and 206 ± 17.2 nM in the 10, 20, 40 and 80 nmol/kg dosing groups, respectively.

#### PKs of NN9056 in obese Göttingen Minipigs

PK parameters were evaluated using a one-compartment model with first-order absorption and first-order elimination (no lag time), which fitted well to the experimental data. Key PK parameters are shown in Table 2, and the plasma concentration–time curves are shown in Supplementary Fig. S2.

**Table 2:** Primary pharmacokinetic parameter estimates from the one-compartmental pharmacokinetic analysis in obese Göttingen Minipigs after subcutaneous dosing of NN9056

Parameter (unit)	Value geometric mean [min;max]
*T*_max_ (h)	3.4 [2.7;5.3]
*C*_max_ (pmol/L)	76,775 [66294;90714]
AUC/dose (h*kg/L)	3091 [3000;3275]
*t*_½_ (h)	128 [107;146]
K01 (1/h)	1.6958 [0.9997;2.2927]
K10 (1/h)	0.005435 [0.004763–0.006492]
V/F (L/kg)	0.05952 [0.05134–0.06983]
Cl/F (L/h/kg)	0.000323 [0.000305;0.000333]

*t*_*½*_ terminal half-life, *K01* absorption rate constant, *K10* elimination rate constant

#### Sub-chronic effects on food intake and BW in obese Göttingen Minipigs

Overall, treatment with NN9056 was well tolerated. During the study, one animal from the NN9056 low-dose group was euthanised on day 85 due to respiratory distress and circulatory failure unrelated to the test compound.

**Fig. 2:**
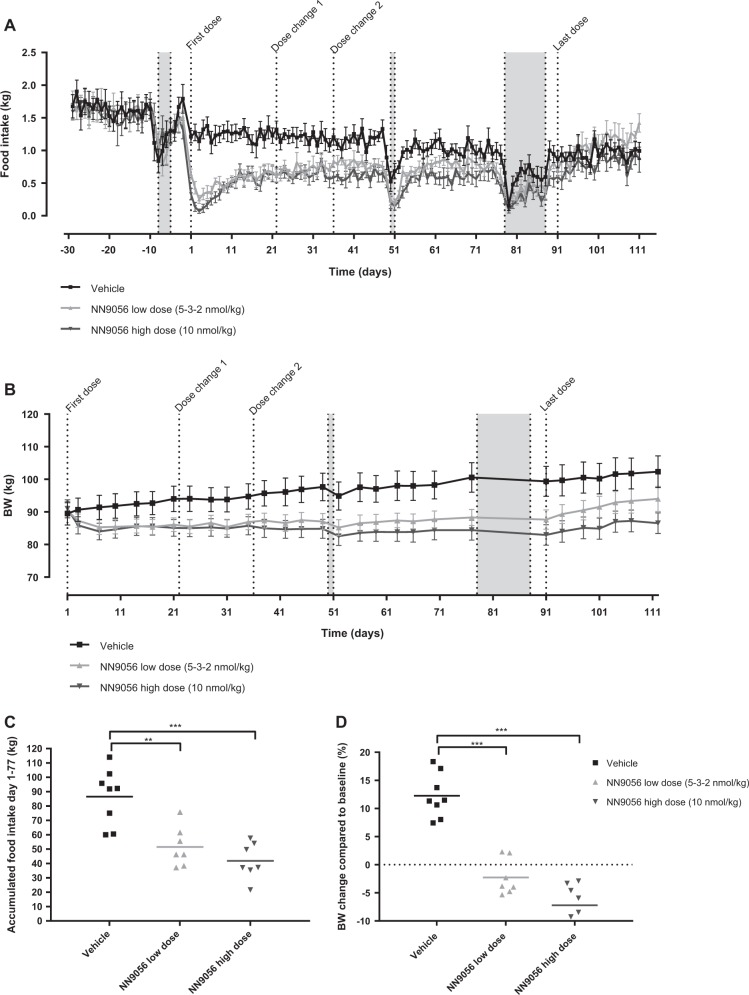
Mean daily food intake (**a**), body weight (BW) development (**b**), accumulated food intake from days 1 to 77 (**c**) and BW change from days 1 to 77 in obese Göttingen Minipigs dosed subcutaneously (s.c.) with either vehicle (black ■, *n* = 8), NN9056 low-dose group (light grey ▲, *n* = 7) or NN9056 high-dose group (dark grey ▼, *n* = 7). Data are presented as mean ± SEM. BW change and accumulated food intake was compared by one-way analysis of variance (ANOVA) followed by Tukey’s multiple comparisons test. **p < 0.01, ***p < 0.001. Grey shading: Periods with anaesthesia/fasting due to dual-energy x-ray absorptiometry (DEXA) scanning or metabolic tests. Dose change 1: dose change from 5 to 3 nmol/kg in the low-dose group. Dose change 2: dose change from 3 to 2 nmol/kg in the low-dose group

The accumulated food intake was decreased in all treated groups compared to vehicle, but with no differences between the two NN9056 groups (41.8 ± 12.6, 51.513.8 and 86.5 ± 19.5 kg in high-dose, low-dose and vehicle groups, respectively, *p* < 0.001 vs. vehicle) (Fig. 2). In parallel with this, there was a BW loss in both the treated groups vs. a BW gain in the vehicle group (−7.2 ± 4.6, −2.3 ± 3.2 and 12.3 ± 3.9% in the high-dose, low-dose and vehicle groups, respectively, *p* < 0.001 vs. vehicle), again with no significant difference between the two NN9056 groups (Fig. 2). The BW change from baseline from days 1 to 77 within each group showed a significant weight increase in the vehicle group (89.5 ± 10.0 vs. 100.6 ± 12.8 kg, *p* < 0.001), a non-significant weight loss in the low-dose group (90.4 ± 6.2 vs. 88.3 ± 6.6 kg) and a significant weight loss in the high-dose group (91.0 ± 7.2 vs. 84.4 ± 8.1 kg, *p* < 0.001).

#### Glucose tolerance and plasma parameters in obese Göttingen Minipigs

**Fig. 3:**
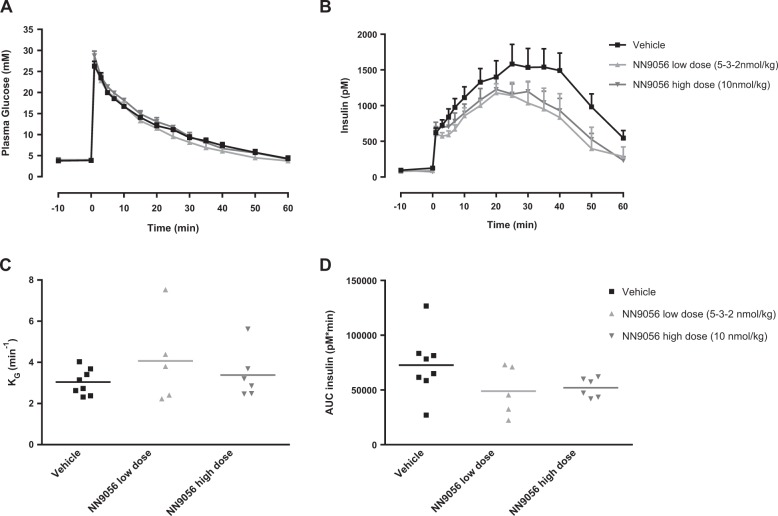
Mean plasma glucose (**a**), mean plasma insulin (**b**), *K*_G_ (**c**) and AUC_insulin_ (**d**) during intravenous glucose tolerance test (IVGTT) in obese Göttingen Minipigs following an IVGTT performed after 12 weeks of subcutaneous (s.c.) treatment with either vehicle (black ■), NN9056 low dose (light grey ▲) and NN9056 high dose (dark grey ▼). Data are presented as mean ± SEM, *n* = 5–8. *K*_G_ and AUC_insulin_ were compared between the groups using one-way analysis of variance (ANOVA) followed by Tukey’s multiple comparisons test. *p* = non-significant for all

During the IVGTT, no significant differences were found between the groups in either glucose clearance (*K*_G_) (3.4 ± 1.2, 4.1 ± 2.1 and 3.0 ± 0.6 min^−1^ in high-dose, low-dose and vehicle groups, respectively) or AUC_insulin_ (52.0 ± 8.9, 48.9 ± 22.7 and 72.7 ± 28.3 nM*min in high-dose, low-dose and vehicle groups, respectively), although AUC_insulin_ tended to be decreased in the two NN9056 groups compared to vehicle (Fig. 3). This indicates a slight improvement in insulin sensitivity, most likely secondary to the weight loss. No statistical analysis was done on glucagon data due to many of the values being below lower level of quantification for the assay (data not shown).

No significant differences between the groups were found in any of the fasting plasma parameters (glucose, insulin, C-peptide, glucagon, total glucagon-like peptide-1 (GLP-1), TBA, leptin, TG, TC, α-amylase and lipase) (Table 3).

**Table 3:** Metabolic parameters in obese Göttingen minipigs evaluated after 12½ weeks of s.c. treatment with either vehicle, NN9056 low dose or NN9056 high dose

Parameter	Vehicle	NN9056 low dose	NN9056 high dose
Glucose (mM)	4.1 ± 0.4	4.0 ± 0.2	4.0 ± 0.2
Insulin (pM)^a^	136 ± 80	78 ± 20	68 ± 9*
C-peptide (pM)	47 ± 28	24 ± 10	24 ± 2
Glucagon (pM)	11.6 ± 3.9	13.5 ± 9.1	11.6 ± 2.3
Total GLP-1 (pM)	34.6 ± 9.7	37.1 ± 9.8	37.6 ± 12.3
TBA (µM)	6.7 ± 3.6	7.8 ± 3.2	9.0 ± 3.4
TG (mM)	0.63 ± 0.35	0.56 ± 0.21	0.41 ± 0.10
TC (mM)	2.3 ± 0.48	2.1 ± 0.49	2.1 ± 0.59
Leptin (ng/ml HE)^**a**^	42.7 ± 11.5	38.6 ± 13.3	47.2 ± 13.3
α-Amylase (U/L)	1570 ± 235	1568 ± 430	1755 ± 404
Lipase (U/L)	8.8 ± 2.1	12.5 ± 7.4	9.4 ± 1.6
Average NN9056 conc. (nM) during the 24 h profile	NA	175 ± 35	1130 ± 18

The groups were compared using one-way ANOVA followed by Tukey’s multiple comparisons test (Kruskal–Wallis followed by Dunn’s multiple comparison test for insulin). *p < 0.05, all other *p* values were non-significant. For C-peptide there was a significant treatment related trend (*p* < 0.05)

*GLP-1* glucagon-like peptide-1, *TBA* total bile acid, *TG* triglycerides, *TC* total cholesterol, *HE* human equivalent, *ANOVA* analysis of variance, NA not available

^a^Data are presented as mean plus or minus SD, *n* = 5–8

Steady-state NN9056 plasma concentration showed an approximate 6-fold difference between the two dose groups (Table 3). Exposure during the 3-week wash-out period is shown in Supplementary Fig. S2.

## Discussion

The present study examined the effect of the long-acting and highly CCK-1 receptor-selective CCK analogue, NN9056, on food intake and BW in obese Göttingen Minipigs.

Results from in vitro receptor-binding and activation assays indicated highly comparable relative affinities and potencies of NN9056 for pig and human CCK receptors. Combined with a low pancreatic expression of CCK-1 receptor mRNA, acceptable acute in vivo tolerability and lack of histopathological signs of pancreatitis after a single, high dose of NN9056 in lean LYD pigs, this confirmed the pig as a relevant model for evaluation of pharmacodynamic effects of CCK analogues. This was further substantiated by a dose dependent acute reduction in food intake of NN9056 in lean LYD pigs.

Consequently, 5 and 10 nmol/kg of NN9056 was dosed once daily in obese Göttingen Minipigs to estimate the effective dose and exposure levels in an obesity model. Both dose levels led to an immediate and significant reduction in food intake, but since the effect on food intake was essentially the same in the two dose groups, the low-dose group was decreased first to 3 nmol/kg and then to 2 nmol/kg, in order to try to obtain differentiation between the two dose levels. Although dose differentiation was achieved, the total accumulated food intake in the two dose groups was not significantly different, and thus maximal efficacy seemed to be obtained after once daily dosing of 3–5 nmol/kg. The reduced food intake lead to a small decrease in BW in both dose groups, which was significantly different from the quite substantial BW gain observed in the vehicle group. These data are in contrast to previously reported efficacy data of a small-molecule CCK-1 receptor agonist showing no effect on BW in obese humans [17]. The lack of efficacy in that study could be related to insufficient exposure, but it has also been proposed to be due to a sub-optimal trial design, with strict caloric restriction preventing the primary appetite-reducing effect to be observed [27]. The half-life of NN9056 was around 130 h in the obese Göttingen Minipigs, and thus full 24-h plasma exposure coverage was obtained throughout the study. This may, together with the *ad libitum* feeding regime, be part of the explanation for the significant and sustained effect on food intake and BW obtained with this CCK analogue.

In contrast to the anti-diabetic effects reported in rodents [3], there were no significant effects on plasma glucose in the Göttingen Minipigs, which could be due to the fact that they are not hyperglycemic (Table 3). However, insulin resistance is present in the model (Christoffersen B, 2018, Insulin resistance in the obese Göttingen Minipig model, personal communication), and a trend for lower fasting insulin and lower insulin secretion during the IVGTT in the NN9056-treated groups compared to vehicle was observed, indicating improved insulin sensitivity secondary to the weight loss.

Treatment with NN9056 was well tolerated, but pathological effects on the pancreas after sub-chronic dosing cannot be completely excluded, since pancreas histology was only evaluated after a single high dose in lean LYD pigs. In contrast, the older, obese Göttingen Minipigs were treated subchronically and furthermore seemed to be more sensitive to the treatment than the young LYD pigs as a 10 nmol/kg dose gave a much stronger effect in the obese Göttingen Minipigs compared to the LYD pigs (Figs. 1 and 2). This difference between the two pig strains may be due to both exposure differences and differences in sensitivity. However, the lack of severe clinical signs, together with lipase and amylase levels within the normal range, indicate that pancreatitis was not an issue even at the exposure levels obtained in the obese Göttingen Minipigs (an average plasma concentration of approx. 1100 nM in the high-dose group).

In conclusion, NN9056, a long-acting CCK-1 receptor-selective analogue, was well tolerated and significantly reduced food intake and BW in obese Göttingen Minipigs during a 13-week study. NN9056 holds a promising potential as monotherapy for obesity or in combination with other appetite-regulating peptides.

## Supplementary information

Supplementary Figure S1

Supplementary Figure S2

Supplementary figure legends

Supplementary Table S1

Supplementary Table S2

Supplementary Table S3

Supplementary Table S4
