# Supplementary figures and images for: Long-acting CCK analogue NN9056 lowers food intake and body weight in obese Göttingen Minipigs
Source: Int J Obes (Lond). 2019 Jun 7;44(2):447–56. doi: 10.1038/s41366-019-0386-0 (PMC6997118; doi:10.1038/s41366-019-0386-0)

**Supplementary Figure S1**

**
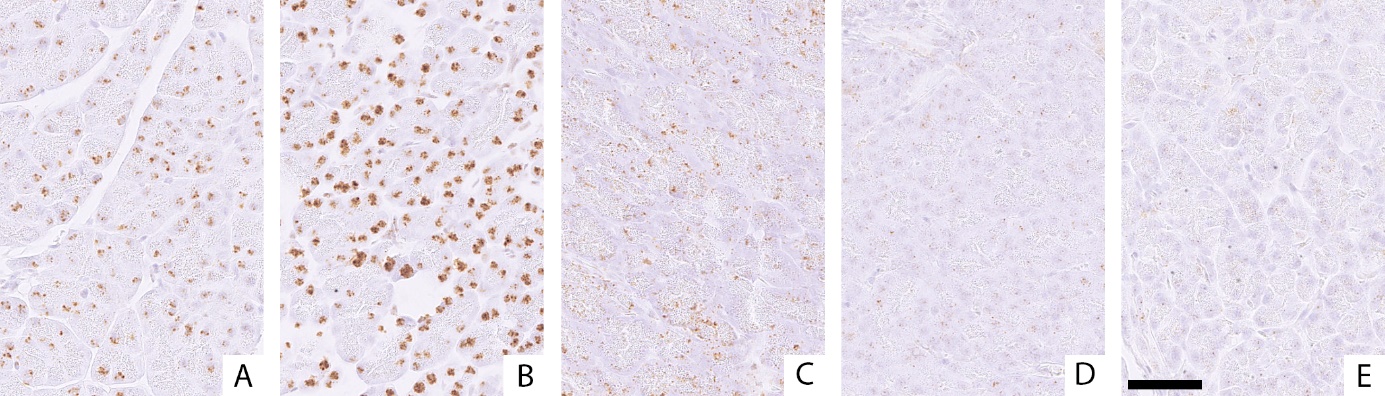
**

Supplement: Supplementary file 1 — Supplementary Figure S1 [file 41366_2019_386_MOESM1_ESM.docx]
