# Supplementary material for: Long-acting CCK analogue NN9056 lowers food intake and body weight in obese Göttingen Minipigs
Source: Int J Obes (Lond). 2019 Jun 7;44(2):447–56. doi: 10.1038/s41366-019-0386-0 (PMC6997118; doi:10.1038/s41366-019-0386-0)
Supplement: Supplementary file 3 — Supplementary figure legends [file 41366_2019_386_MOESM3_ESM.docx]

**Supplementary Figure S1**

Detection of CCK-1 receptor mRNA in formalin fixed, paraffin embedded pancreas, RNA in situ hybridization using RNAscope®. A. Mouse, B. Rat, C. Dog, D. Pig and E. Human. Scalebar: 50µm.

**Supplementary Figure 2**

Plasma concentration-time profiles after a single dose of NN9056 (5 nmol/kg) (A, B). Measured plasma concentrations during the 24 h profile and the following 3 weeks wash-out period (C, D) in obese Göttingen minipigs dosed s.c. with either NN9056 low dose (light grey ▲, n=7) or NN9056 high dose (dark grey ▼, n=7). T=0 corresponds to the time of the last dose on day 91. Linear scale (A, C), log scale (B, D). Data are presented as mean ± SEM.
