# Supplementary material for: Long-acting CCK analogue NN9056 lowers food intake and body weight in obese Göttingen Minipigs
Source: Int J Obes (Lond). 2019 Jun 7;44(2):447–56. doi: 10.1038/s41366-019-0386-0 (PMC6997118; doi:10.1038/s41366-019-0386-0)
Supplement: Supplementary file 4 — Supplementary Table S1 [file 41366_2019_386_MOESM4_ESM.docx]

**Supplementary Table S1**

**Power Calculations**

| **Study** | **Power calculation** |
| --- | --- |
| Acute food intake in lean LYD pigs  Acute tolerability in lean LYD pigs | Based on the variation in similar studies 3-4 animals per group are necessary to detect a reduction in food intake of ~25 % compared to vehicle given that multiple comparisons are required (2 treatment groups compared to vehicle, α=0.05, β=0.8). |
| Pharmacokinetic evaluation in obese Göttingen minipigs | Based on the standard deviation in similar studies performed in Göttingen minipigs, the estimated precision on the primary parameters of interest (clearance, volume of distribution and t½) is from 0.8-1.20 times the calculated mean, which was considered to be sufficient in this study. |
| Obese Göttingen minipigs subchronic study | Based on the variation in similar studies in obese Göttingen minipigs, 6 animals are necessary to detect a difference in BW of 15 % compared to vehicle and 6 animals are required to detect a reduction in food intake of 50 % compared to vehicle given that multiple comparisons are required (2 treatment groups compared to vehicle, α=0.0274, β=0.8). Based on experience from previous long term studies it may sometimes be necessary to exclude an animal from the groups due to either a too great increase in body weight (particularly in the vehicle group), lameness or similar model related problems and sometimes catheter dysfunction is observed in a few animals precluding their participation in metabolic tests, and hence 7-8 animals were included in the groups from the beginning to ensure enough power at end study evaluations |

**Data exclusions**

In general, all analysis results below LLOQ were excluded.

| **Study** | **Data exclusion** |
| --- | --- |
| Acute food intake in lean LYD pigs | One animal was excluded from the 40 nmol/kg group. The animal moved around during the s.c. dosing procedure resulting in unknown dosing volume. Food intake measurements at 24h and 48h in one animal in the vehicle group were excluded due to clotting of the feeding tube resulting in invalid food intake data. |
| Acute tolerability in lean LYD pigs | TBA measurements from one vehicle animal were excluded due to unusually high values (15 fold higher at 24 h) indicating a mistake in the dilution of the blood sample. |
| Pharmacokinetic evaluation in obese Göttingen minipigs | No data exclusions |
| Obese Göttingen minipigs subchronic study | One animal in the low dose group was euthanised on day 85 of the study; data until day 83 has been included.  In the IVGTT, one animal in the NN9056 low dose group and one animal in the NN9056 high dose group had dysfunctional catheters and were not included in the test. During the exposure profile and for the fasting plasma sample, one animal in the NN9056 high dose group had dysfunctional catheter and no samples could be obtained.  One animal in the low dose group and one animal in the high dose group had unusual low amylase values (16-20 fold lower than the other animals in the respective group) and the lipase values in both these animals were below LLOQ. This could indicate a mistake in the blood sampling procedure with possible sample dilution, and the values for amylase (and also lipase) were therefore excluded. |
