# Supplementary material for: Long-acting CCK analogue NN9056 lowers food intake and body weight in obese Göttingen Minipigs
Source: Int J Obes (Lond). 2019 Jun 7;44(2):447–56. doi: 10.1038/s41366-019-0386-0 (PMC6997118; doi:10.1038/s41366-019-0386-0)
Supplement: Supplementary file 5 — Supplementary Table S2 [file 41366_2019_386_MOESM5_ESM.docx]

**Supplementary Table S2**

**Assay information**

| **Analyte** | **Assay Details** | **Assay Sensitivity/ LLOQ** |
| --- | --- | --- |
| NN9056 | The plasma samples (including quality control and standard curve samples used for quantitation of unknowns and prepared from blank plasma spiked with CCK derivative at a concentration range of 0.5-1000 nM) were protein precipitated using three volumes of methanol (including internal standard NNC0368-0000-0108) and centrifuged (16000g, 4°C, 30 min). The supernatants (25 µL) were injected into the chromatographic system (TurboFlow Transcend 1250 & 10 valve VIM, Thermo Fisher Scientific) which consisted of an initial Turboflow Cyclone purification column 0.5 x 50 mm (Thermo Fischer Scientific) and an eluting Aeris peptide 3.6 µm XB - C18 column 2.1 x 50 mm (Phenomenex) kept at 60°C. The CCK derivative was eluted using a chromatographic gradient system with mobile phases consisting of water/acetonitrile 90/10 v/v% with 1 v/v% formic acid (mobile phase A) and acetonitrile with 1v/v% formic acid (mobile phase B). The first 0.25 minute gradient started at 0% mobile phase B and ended at 70% mobile phase B followed by a second 2.17 min gradient starting at 70% mobile phase B and ending at 75% mobile phase B with a flow rate of 0.4 ml/min. The CCK derivative was detected and quantified after on-line infusion of the LC flow to the LTQ OrbiTrap Discovery mass spectrometer (Thermo Fischer Scientific) equipped with an electrospray interface operated in negative mode, ESI. | 1000 pM |
| TBA, TG, TC, pancreas lipase and pancreas α-amylase | TBA, TG, TC, pancreas lipase and pancreas α-amylase were measured on a Cobas® 6000 autoanalyzer (Roche Diagnostics GmbH, Mannheim, Germany) according to the manufacturer’s instructions. | TBA: 1 µM  TG: 0.1 mM  TC: 0.1 mM  α-amylase: 3 U/l  Lipase: 3 U/l |
| **Analyte** | **Assay Details** | **Assay Sensitivity/ LLOQ** |
| Plasma glucose | Ten (10) µL of plasma was transferred into 500 µL EBIO solution and measured on a Biosen auto analyzer (BIOSEN S_Line, EKF Diagnostics, Cardiff, UK) according to the manufacturer’s instructions. | 0.5 mM |
| Insulin | Insulin was measured using Luminescence Oxygen Channeling Immunoassay (LOCI/ AlphaLisa) by applying a mixture of biotinylated mAb Oxi-005 and mAb HUI-018 -conjugated acceptor-beads. | 3 pM |
| C-peptide | C-peptide content was determined using LOCI/ AlphaLisa assay by applying a mixture of biotinylated mAb 4F16A6 and mAb 1F341A -conjugated acceptor-beads. | 15 pM |
| Glucagon | Glucagon content was determined using LOCI/ AlphaLisa by applying a mixture of biotinylated mAb GLU 2F7 and mAb GLU 1F120 conjugated acceptor-beads | 4 pM |
| GLP1 (total) | Total GLP1 content was analysed using LOCI/ AlphaLisa by applying a mixture of biotinylated mAb HYP-147 and mAb GLPa-1F5 conjugated acceptor-beads. | 20 pM |
| Leptin | Plasma leptin was analysed using a commercial kit from Milipore (Multi-Species Leptin RIA Kit, Cat no. XL-85K, EMD Milipore Corporation). The analysis was performed according to the manufacturer´s instructions, and since the antibody was raised against human leptin and has an unknown cross-reactivity with leptin from other species, the results are given in ng/mL human equivalents. | 1 ng/ml (human equivalent) |
