# Supplementary material for: Long-acting CCK analogue NN9056 lowers food intake and body weight in obese Göttingen Minipigs
Source: Int J Obes (Lond). 2019 Jun 7;44(2):447–56. doi: 10.1038/s41366-019-0386-0 (PMC6997118; doi:10.1038/s41366-019-0386-0)
Supplement: Supplementary file 6 — Supplementary Table S3 [file 41366_2019_386_MOESM6_ESM.docx]

**Supplementary Table S3**

**Composition of Zoletil mixtures used for anaesthesia of LYD pigs and obese Göttingen minipigs and of the stabilisation buffer used for blood samples in the obese Göttingen minipigs.**

| **Composition of Zoletil mixture for LYD pigs** | 125 mg zolazepam and 125 mg tiletamin (Zoletil ® 50 Vet., ChemVet, Denmark), ketamin (1.25 ml Ketaminol® Vet. 100 mg/ml, Intervet, Denmark), medetomidin (6.5 ml Cepetor Vet, Medetomidin 1 mg/ml, ScanVet Animal Health A/S) and buthorphanol (2.5 ml Torbugesic®, 10 mg/ml, Scanvet, Denmark). |
| --- | --- |
| **Composition of Zoletil mixture for the obese Göttingen minipigs** | 125 mg zolazepam and 125 mg tiletamin (Zoletil ® 50 Vet., ChemVet, Denmark), ketamin (1.25 ml Ketaminol® Vet. 100 mg/ml, Intervet, Denmark), xylacin (6.5 ml Rompun Vet, 20 mg/ml, Bayer A/S, Denmark) and buthorphanol (2.5 ml Torbugesic®, 10 mg/ml, Scanvet, Denmark). |
| **Composition of stabilisation buffer for blood samples in obese Göttingen minipigs** | Stabilisation buffer was prepared by adding 40 mg of Pefabloc SC (Roche Diagnostics GmBH, Mannheim, Germany ) to 10 ml of a buffer containing 317 mM K_3_EDTA (Sigma-Aldrich, Steinheim, Germany), 10.000 KIU/mL aprotinin (Nordic Pharma, Berkshire, United Kingdom) and 0.4 mM valine-pyrrolidide (pre-pared at Novo Nordisk A/S). To each tube also 20 µl of Protease inhibitor cocktail P.I.C. (Sigma-Aldrich, Steinheim, Germany) was added. |
