# Supplementary material for: Long-acting CCK analogue NN9056 lowers food intake and body weight in obese Göttingen Minipigs
Source: Int J Obes (Lond). 2019 Jun 7;44(2):447–56. doi: 10.1038/s41366-019-0386-0 (PMC6997118; doi:10.1038/s41366-019-0386-0)
Supplement: Supplementary file 7 — Supplementary Table S4 [file 41366_2019_386_MOESM7_ESM.docx]

**Supplementary Table S4**

**Pancreatic lipase, pancreatic α-amylase and total bile acids in the pre-dose sample and 48 h post-dose sample in lean LYD pigs dosed with 10 and 100 nmol/kg NN9056 (tolerability study). Mean±SD, n=4.**

| **Parameter** | **Vehicle**  **Pre-dose** | **Vehicle**  **48 h** | **10 nmol/kg**  **NN9056**  **Pre-dose** | **10 nmol/kg**  **NN9056**  **48 h** | **100 nmol/kg**  **NN9056**  **Pre-dose** | **100 nmol/kg**  **NN9056**  **48 h** |
| --- | --- | --- | --- | --- | --- | --- |
| **TBA**  **(µM)** | 2.9±0.3^#^ | 4.1±3.2^#^ | 3.0±0.6 | 3.7±0.8 | 2.8±0.6 | 3.1±1.3 |
| **α-amylase (U/L)** | 2333±302 | 2254±335 | 1859±221 | 1923±488 | 2488±522 | 2238±291 |
| **Lipase (U/L)** | 5.6±0.6 | 5.6±0.3 | 5.3±0.6 | 6.4±0.9 | 5.7±0.7 | 6.1±0.2 |

The groups were compared using one-way ANOVA followed by Sidak's multiple comparisons test. All p-values were non-significant (p<0.05). ^#^n=3.
